# Effects of berberine hydrochloride on immune response in the crab *Charybdis japonica*

**DOI:** 10.1186/s12864-022-08798-w

**Published:** 2022-08-11

**Authors:** Tian-heng Gao, Ming-ming Han, Hui Zhou, Chen-xi Zhu, Ying Yang, Zakaria Zuraini, Yan-Xia Guo, Qi-chen Jiang

**Affiliations:** 1 Institute of Marine Biology, College of Oceanography, Hohai University, Nanjing, 210024 China; 2 Biology Program, School of Distance Education, Universiti Sains Malaysia, 11800 Minden, Penang, Malaysia; 3 Jiangsu Key Laboratory for Molecular and Medical Biotechnology, College of Life Sciences, Nanjing Normal University, Nanjing, 210023 China; 4 Freshwater Fisheries Research Institute of Jiangsu Province, 79 Chating East Street, Nanjing, 210017 China

**Keywords:** berberine hydrochloride, *Charybdis japonica*, Transcriptome sequencing, Immune response

## Abstract

Berberine hydrochloride is the main effective component of Coptis spp. used in Chinese herbal medicine and its underlying molecular mechanisms, responsible for inducing effects in crustacean species, are not fully understood. In this study, the molecular response of the crab *Charybdis japonica* to berberine hydrochloride exposure was studied using transcriptome sequencing. The survival rate, gene expression and activities of several immune enzymes were measured after berberine hydrochloride treatments, with or without injection of the pathogenic bacterium *Aeromonas hydrophila*. A total of 962 differentially expressed genes (464 up-regulated and 498 down-regulated) were observed during exposure to 100 mg/L of berberine hydrochloride and in the control group after 48 h. Enrichment analysis revealed that these genes are involved in metabolism, cellular processes, signal transduction and immune functions, indicating that exposure to berberine hydrochloride activated the immune complement system. This bioactive compound simultaneously activated fibrinogen beta (FGB), fibrinogen alpha (FGA), alpha-2-macroglobulin (A2M), kininogen (KNG), fibrinogen gamma chain (FGB), alpha-2-HS-glycoprotein (AHSG), caspase-8 (CASP8), cathepsin L (CTSL), adenylate cyclase 3 (Adcy3) and MMP1. Its action could significantly increase the survival rate of the crabs injected with *A. hydrophila* and promote the activity of LZM, Caspas8, FGA, ACP and AKP in the hepatopancreas. When *A. hydrophila* was added, the neutralization of 300 mg/L berberine hydrochloride maximized the activities of Caspas8, LZM, ACP and AKP. Our results provide a new understanding of the potential effects of berberine hydrochloride on the immune system mechanisms in crustaceans.

## Introduction

Berberine hydrochloride is the *hydrochloride* form of the natural isoquinoline alkaloid, and is the main active ingredient of several commonly used Chinese herbal medicines, such as those derived from Coptis and Phellodendron plants. It has been shown to have antibacterial [1] and antioxidant properties both in in vitro and in vivo experiments [2], and is considered as a potential biological drug that can replace synthetic antibiotics [3]. However, little information is known about its effects on crustacean species.

Presently, bacterial infections are the leading cause of crab diseases, representing an important limiting factor for the entire crab aquaculture industry [4, 5]. According to reports, several bacterial and viral pathogens regularly infect marine crabs [6], among them, are the gram-negative bacteria *Vibrio alginolyticus* and *Aeromonas hydrophila*, that severely affect crab populations causing multiple infections and leading to great economic losses each year [7].

In crabs, the nonspecific immunity is the most important defense against microbial infection. The hepatopancreas, together with body fluids and cell response, controls immune resistance against viruses and bacteria, and it is therefore, the target organ for treatments aimed at improving the response to infections [8–10]. In recent years, transcriptome sequencing has become an effective method for evaluating immune responses in aquatic animals, providing valuable data on their key mechanisms and functions [11]. For example, transcriptomics technology has revealed the biological mechanism behind multiple pathogenic microorganisms affecting crustaceans, such as WSSV and Vibrio species. Many immune-related genes and polymorphic microsatellite markers have been identified in crabs [12–14], providing important genetic information, essential for further understanding the molecular mechanisms of innate immunity.

The Japanese crab, *Charybdis japonica*, belongs to the Portunus family and is a common species with considerable commercial value widespread in Southeast Asia [15, 16]. It typically inhabits benthic estuarine environments, characterized by both fine and muddy sand, and can withstand low oxygen concentrations, a wide range of salinity and large temperature fluctuations [17, 18]. In this study, a comparative transcriptome analysis was conducted to identify differentially expressed genes that could potentially emerge in this species under the action of berberine hydrochloride. Subsequently, the variation of gene expression associated with the related immune enzyme activity after Vibrio treatment was measured, and the regulatory mechanisms of immunity were analyzed. Our research aimed to provide a reference for understanding the molecular mechanisms behind the effects of berberine hydrochloride in crustaceans, and to promote the application of this bioactive compound in aquaculture.

## Materials and methods

### Experimental animals

A total of 580 *Charybdis Japonica* adult crabs (body weight = 80 ± 2.4 g), collected from the South China Sea, were randomly placed in 29 aquariums containing artificial seawater and equipped with a cyclic exposure system (specific conditions were: salinity of 28 psu, temperature of 25 ± 1 ºC, pH 7.5 ± 0.5, a dissolved oxygen concentration of approximately 5.0 mg L^−1^ and a 12 h light/dark photoperiod).

### Berberine hydrochloride treatment

In this experiment, one berberine hydrochloride (HPLC ≥ 98%, Vicky Biotechnology Co., Ltd.) treatment group and one control group were set every triplicate. Before the treatment, all the crabs were cultured for two weeks to adapt to the laboratory environment and have a 24 h starvation treatment. 100 mg/L of berberine hydrochloride was administered to the treatment group for a 48 h exposure test, crabs were selected randomly from both the experimental control groups, and hepatopancreas samples were removed for RNA isolation.

### Bacterial infection test

Samples of *Aeromonas hydrophila* were kindly provided by Shanghai Ocean University (China). After two weeks, the *C. japonica* were not fed for 24 h before the experiment. Water quality remained at the same level as acclimation. The 8 treatments were: *C. japonica* treated with 0, 100, 200, and 300 mg/L of berberine hydrochloride with and without injected with 10^5^ CFU/L of *A. hydrophila*. According to the preliminary experiment, 300 mg/L is harmless to *C. japonica*. *C. japonica* were kept in tanks containing 0, 100, 200 and 300 mg/L berberine hydrochloride in triplicates, and 48 h later, 10^5^ CFU/L of *A. hydrophila* was injected into the fourth leg in half of the crabs. According to the preliminary experiment, *A. hydrophila* 10^5^ CFU/L could cause death in *C. japonica* after 24 h. After the injection of *A. hydrophila*, the survival rate was calculated (starting at 0 h) for different time periods after 24, 96 and 144 h.

### Sample collection

After 24 h injection, 12 crabs were selected randomly, and their hepatopancreas was removed and preserved on ice until further analysis. The hepatopancreas samples of three individuals in each group were mixed in equal amounts. Total RNA was isolated using a TRIzol reagent kit (Invitrogen, USA) following the manufacturer's instructions. An Agilent 2100 Bioanalyzer was used to determine RNA integrity and quality. After extracting total RNA and digesting the DNA with DNase, the eukaryotic mRNA was enriched using magnetic beads with Oligo (dT). A fragmentation reagent was added to break mRNA into short fragments, which were used as templates. Subsequently, one-strand cDNA was synthesized using six-base primers, and double-stranded cDNA was synthesized. After the double-stranded cDNA was purified and repaired, fragment size was selected, and finally PCR amplification was performed. The library was constructed using an Agilent 2100 bioanalyzer (whose quality had been previously evaluated) and an Illumina HiSeqTM 2500 sequencer was used to generate 125 bp or 150 bp paired-end sequence data.

### Data pre-processing, quality control and assembly

Quality control was assessed in Trimmomatic [19] and joints were removed; then low-quality bases and N-bases were filtered out, and only high-quality clean reads were retained [20]. The paired-end splicing method in Trinity (version: 2.4) was used to splice clean reads and obtain transcript sequences: the longest one was selected as Unigene based on sequence similarity and length.

### Transcriptome quality control, de novo assembly and functional annotation

The original reads were firstly filtered in FASTQ format, and then those containing sequencing adapters were removed, together with nucleotides that were unknown (N than N 10%) or had low quality (quality score ≤ 5), in order to obtain clean reads. At the same time, their Q30 and GC contents were calculated. The remaining high-quality reads were used for downstream analysis. Based on different databases, including non-redundant protein sequences (Nr), non-redundant nucleotides (Nt), Swiss-Prot, Orthologous group (COG), Gene ontology database (GO) and the Kyoto Encyclopedia of Genes and Genomes (KEGG), a blastx similarity search was performed on all unigenes, with a threshold of e < 1e-5. The GO annotation of Unigene was determined, and finally Unigene was compared to the KEGG [21] database to obtain access information.

### Unigene quantification, differential Unigene screening, functional enrichment and cluster analysis

Unigene’s FPKM [22] and count were obtained using bowtie2 [23] and express [23] software. In particular, the latter was used to obtain the number of Unigene reads corresponding to each sample [24]. The “estimateSizeFactors” function of DESeq [25] in the R package was used to standardize the data, and the “nbinomTest” function was used to calculate the p-value and fold change value for ranking differentially expressed genes. The Unigene reads with a p value less than 0.05 and with a multiple of difference greater than 2, were selected and an enrichment analysis of the differentially expressed Unigenes, derived from the GO and KEGG databases [22], was performed to determine the biological functions or pathways that they mainly affect. At the same time, unsupervised hierarchical clustering of Unigenes was performed, and their expression pattern among different samples was displayed using a heat map.

### Validation of RNA sequencing and gene expression

To test the reliability of RNA-Seq results, nine candidate genes related to oxidative stress and immune response were selected for qRT-PCR verification. The expression of these genes at different concentrations of berberine hydrochloride, with or without *A. hydrophila,* was also tested. The design of the primers was based on sequences obtained from the RNAseq and is shown in Table 1. Total RNA was extracted from the hepatopancreas of the control and treatment groups. The reverse transcription of RNA was performed using the PrimeScript RT kit with gDNA eraser (Takara, Nanjing, China) following the kit’s standard protocols. Real-time PCR was conducted in three replicates using reactions containing 10 µL of SYBR mixture, 0.6 µL of each primer, 7.6 µL of ddH_2_O, and 1.2 µL of cDNA template. The relative gene expression level was evaluated using the 2^ΔΔ^CT method.

**Table 1:** Primers designed for *Charybdis japonica* and used for real-time PCR analysis

gene	Forward primer (5'- > 3')	Reverse primer (5'- > 3')	Size (bp)
FGB	CAATGGTTTGCTGGTTGGTCA	AACGCTGGATCTTCTCACGGA	143
AHSG	GGTCATCTTGGGAATTACCTT	TTTTGCTGTCACTTTCGTCTC	109
FGA	CTGAAGTCGCAGGTCAAAGAT	CCACCGTGAACTCACTGTAGC	115
A2M	GCCAACAGACAACGCCTATGA	ACTGAGGGACTCGCACAACCA	80
Casp	CCGAAGTAGTGAAGAAATGGC	CGGTCTGATGTAACGAAGGTA	93
FGG	GGCTAACGAGAAACGAATAACT	CTTTGCCTGTGAATGTCTGAAT	110
KNG	TCCTCTAAAGTTGCCCCTTGT	ACCACCTGTTTTGTTGCTGAT	164
Adcy3	GGGCAAGGGTGAACTAATGAC	GCACGGGAATAGTCCAGTGAC	90
MMP1	TTGATGGACCTGGAGGAAAT	AGCCGCAACACGATGTAAGT	131
Beta -actin	CTGCGGAATCCACGAAAC	GTCAGCAATGCCAGGGTA	121

### Enzyme activity

The hepatopancreas was rinsed with ice-cold physiological saline solution. About 0.1 g of hepatopancreas tissue was homogenized with the solution (hepatopancreas/total extract mass¼10%) and the homogenate was centrifuged at 3000 rpm for 10 min at 4 ℃. The supernatant was used for biochemical assays. The activity of the following enzymes, acid phosphatase (ACP), alkaline phosphatase (AKP), catalase (CAT), lysozyme (LZM), fibrinogen alpha chain (FGA) and Caspase8, was determined using commercial test kits (Nanjing Jiancheng Institute of Biotechnology, Nanjing, China) in accordance with the manufacturer's instructions.

### Data analysis

All data were presented as the mean ± SE. To determine the differences between control and treatment groups, one-way analysis of variance (ANOVA) and Tukey’s test were performed. A *P* < 0.05 was considered significant.

## Results

### FPKM value distribution of Unigene

As shown in Table 2, the hepatopancreas samples of both the control and experimental groups were subjected to high-throughput transcriptome Illumina sequencing; four replicate samples were sequenced for each group. The average number of original reads after sequencing was 53,737,002, and the average number of clean reads obtained after filtering was 52,435,205. The average effective base percentage was 94.74%, showing 93.82% of bases with a phred value greater than 30 in raw bass.

**Table 2:** Quality assessment of sequencing data prior to processing

Sample	raw_reads	clean_reads	clean_bases	valid_bases	Q30	GC
BH1_1	53,055,766	51,874,260	7,576,975,390	95.21%	94.01%	49.32%
BH1_2	54,761,130	53,474,898	7,791,927,013	94.86%	93.83%	49.24%
BH1_3	54,462,306	53,114,956	7,761,807,684	95.01%	93.78%	48.72%
BH1_4	53,919,386	52,562,248	7,648,127,562	94.56%	93.64%	48.65%
CK1	54,458,976	53,249,998	7,744,315,340	94.80%	93.90%	49.21%
CK2	50,955,922	49,544,232	7,149,461,766	93.54%	93.74%	49.66%
CK3	54,127,462	52,845,974	7,696,579,447	94.80%	93.88%	48.94%
CK4	54,155,066	52,815,070	7,726,421,293	95.11%	93.77%	49.01%

### Unigene expression, volume annotation and statistics

Unigene expression was calculated using the FPKM method, which considers the number of fragments per million of a certain Unigene per kilobase length. The average value of the hepatopancreas median in the control and treatment groups was 1.98. BH1-3 with the smallest average value of FPKM was 13.8121, and the highest CK3 value was 14.93026. The maximum FPKM value and standard deviation for CK1 was 23,433.73 and 166.8806, respectively. The FPKM sum of eight *Charybdis japonica* samples amounted to 5,781,915.

### Results of principal component analysis (PCA analysis) and Unigene statistical diagram of differential expression

**Fig. 1:**
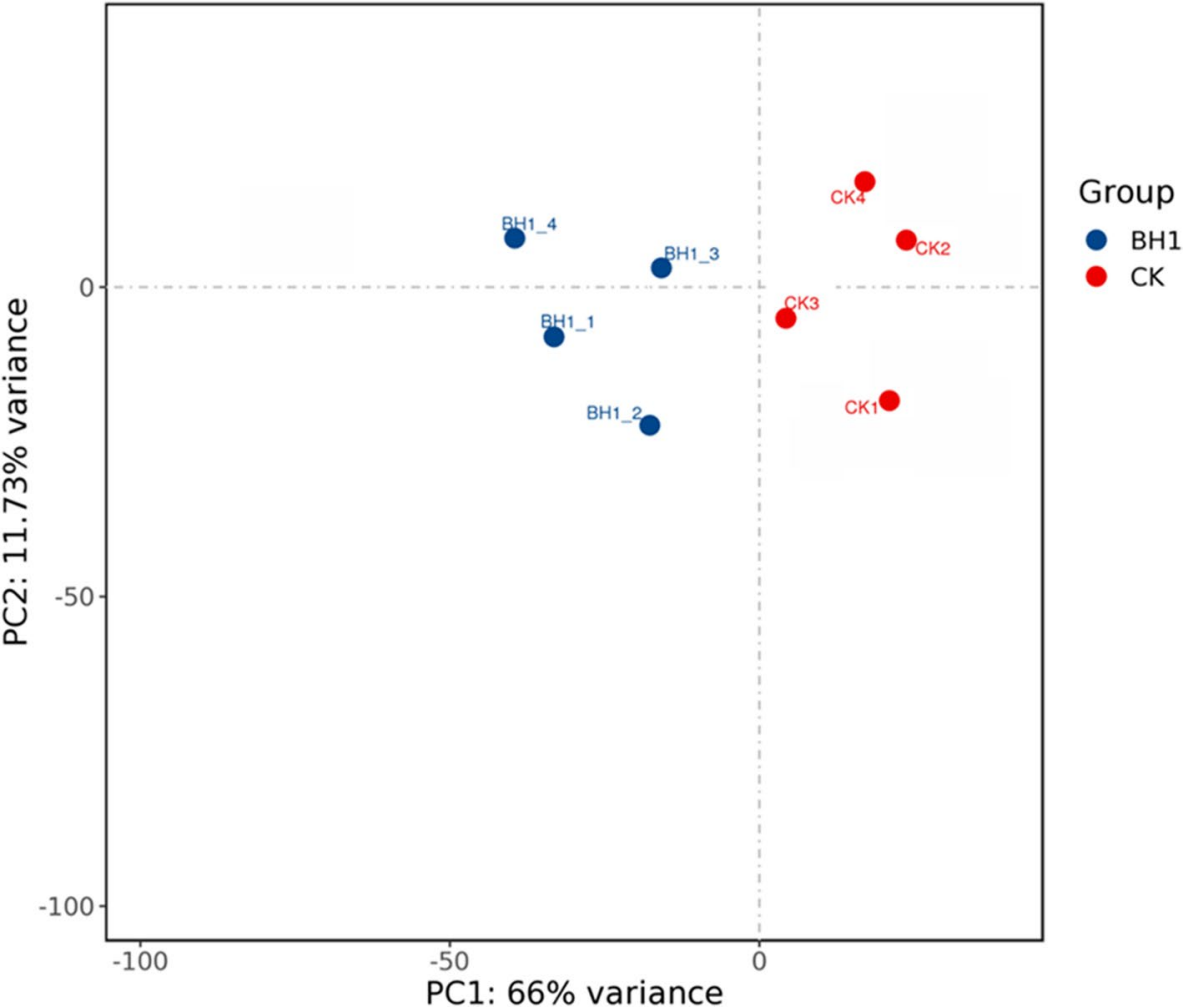
Principal component analysis (PCA analysis) using global expression of unigenes. Blue circles show *Charybdis japonica* exposed to 100 mg/L of berberine hydrochloride and red circles indicate control samples

**Fig. 2:**
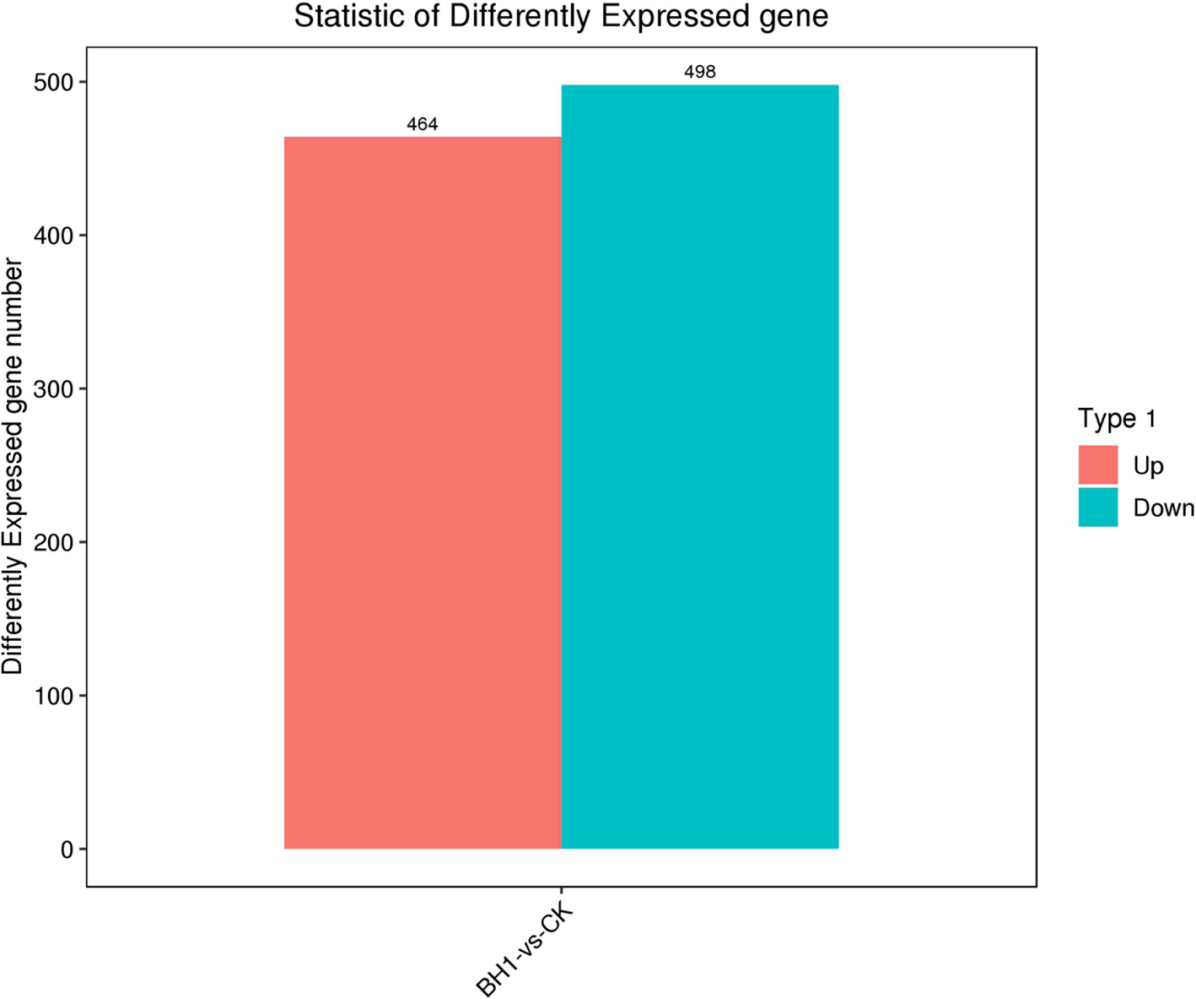
Comparison of the number of differentially expressed genes in treatment and control groups. The red and blue columns indicate the number of upregulated and downregulated genes, respectively

The relationship between the experimental and control group samples was explored through principal component analysis (PCA) (Fig. 1), which showed that BH1-1, BH1-2, BH1-3, and BH1 in the BH1 *Charybdis japonica* sample presented different dimensions. The sample clustering distance of BH1-4 and that of CK1, CK2, CK3, and CK4 in BCK, were similar in the two-dimensional space. The results showed that the control and experimental groups had multiple up-regulated and down-regulated differences between the samples, corresponding to 464 and 498 Unigenes, respectively; the total number of significantly different expressions of Unigenes was 962 (Fig. 2).

### Results of KEGG enrichment analysis

**Fig. 3:**
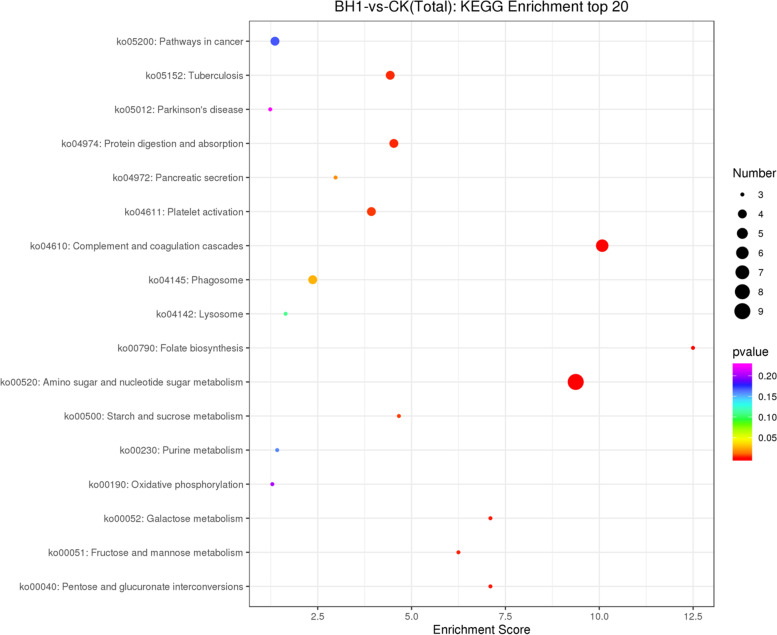
Top 20 pathways in the KEGG enrichment analysis with differentially expressed genes. The smaller the enrichment *p *Value < 0.05

Differentially expressed genes (Fig. 3) in complement and coagulation cascades with innate immune function include fibrinogen beta (FGB), fibrinogen alpha (FGA), alpha-2-macroglobulin (A2M), kininogen (KNG) and the fibrinogen gamma chain (FGB). In particular, adenylate cyclase type 3 (ADCY3), fibrinogen beta (FGB), fibrinogen alpha (FGA) and the fibrinogen gamma chain (FGB) are involved in platelet activation. Lysophospholipase III (LYPLA3) and cathepsin L (CTSL) in lysosomes with innate immune function were significantly changed, and Niemann-Pick C2 protein (NPC2) was down-regulated. NADH-ubiquinone oxidoreductase chain 2 (ND2) in oxidative phosphorylation, alkaline phosphatase activity and molybdenum cofactor biosynthesis protein 1 (Mocs1) in galactose metabolism were all up-regulated. In amino sugar and nucleotide sugar metabolism, UDP-glucose 4-epimerase (Gale), UDP-glucuronate decarboxylase (uxs1), phosphoglucomutase-2 (PGM2) and hydrolase activity CHIT1 were up-regulated. In the same metabolisms, chitinase (Chia) was down-regulated. Fructose and mannose metabolism and Starch and sucrose metabolism genes are also significantly changed.

### Differential expression of Unigenes in the KEGG results

**Fig. 4:**
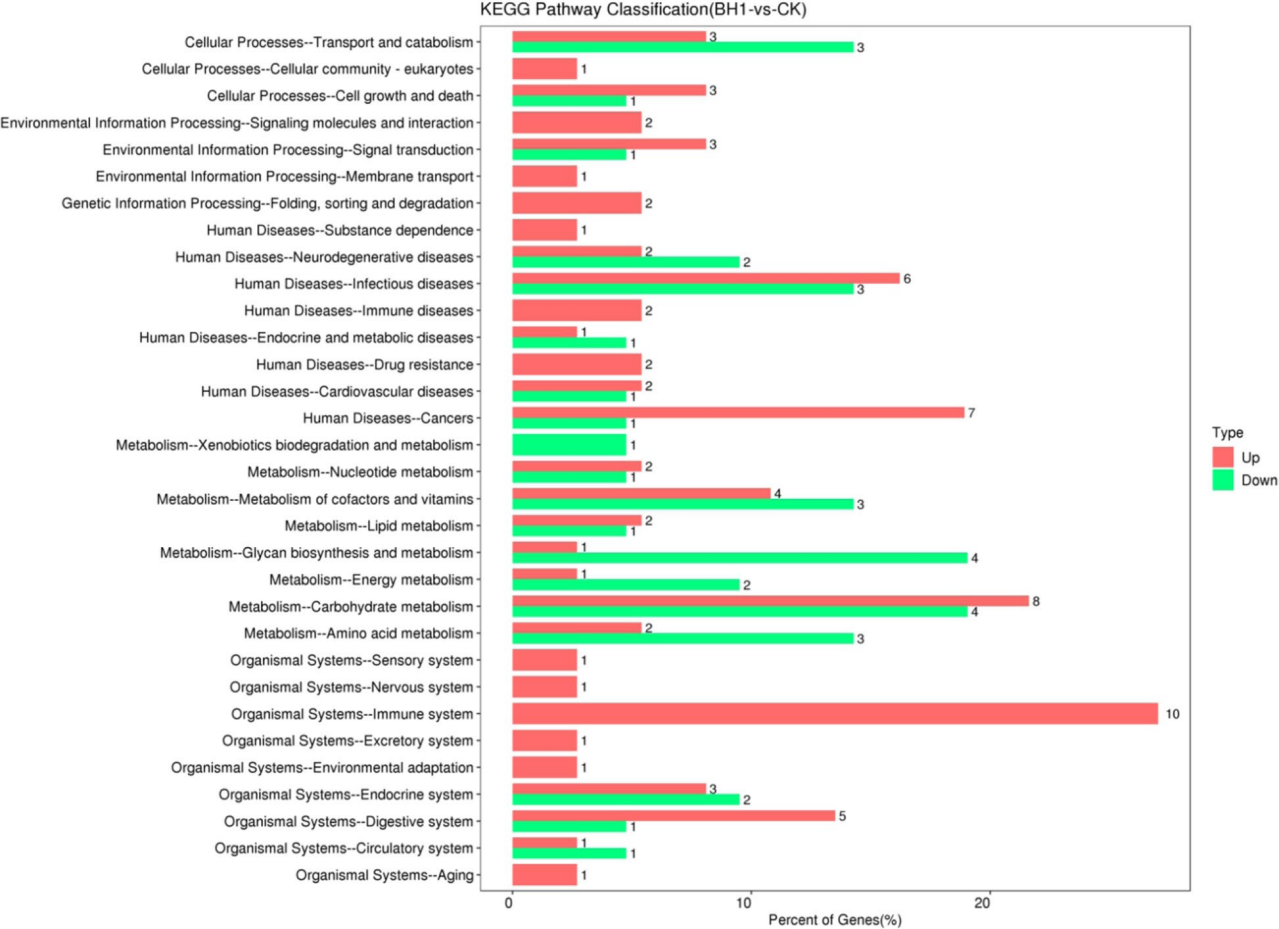
Significantly enriched pathways containing differentially expressed genes (DEGs). The red and green columns indicate the number of upregulated and downregulated genes, respectively

The up-regulation and down-regulation of differentially expressed Unigenes are presented in the KEGG level 2 distribution diagram(Fig. 4) One gene in environmental adaptation was on the rise. Nucleotide metabolism had two increasing genes and one decreasing gene. Glycan biosynthesis and metabolism had one up-regulated gene and four down-regulated genes. Lipid metabolism had two up-regulated genes and one down-regulated gene. Because *C. japonica* can only rely on the innate immune system to respond to viruses or bacteria, the focus of the present study was precisely the immune system, which presented 10 up-regulated genes—FGA, FGB, FGG, KNG, A2M, MMP1, AHSG, caspase-8 (CASP8), cathepsin L (CTSL) and adenylate cyclase 3 (Adcy3)— and five down-regulated genes—egr, adenosine kinase **(**Adk), chia, angiotensin-converting enzyme (ACE) and surfactant protein-A1 **(**SFTPA1).

### Gene transcription level

**Fig. 5:**
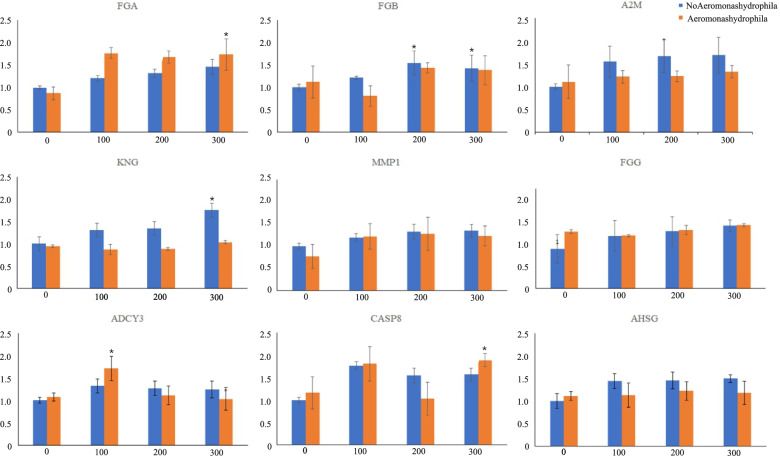
Survival rate of *Charybdis japonica* at 24 h, 96 h, and 144 h after being infected with *A. hydrophila:* CK (control group) AH (*A. hydrophila* injection of 10^5^ CFU/L), BH1 (exposure to 100 mg/L of *berberine hydrochloride*), AHBH1(*A. hydrophila* injection of 10^5^ CFU/L and exposure to 100 mg/L of *berberine hydrochloride*), BH2 (exposure to 200 mg/L *berberine hydrochloride*), AHBH2 (*A. hydrophila* injection of 10^5^ CFU/L and exposure to 200 mg/L of *berberine hydrochloride*), BH3 (exposure to 300 mg/L of *berberine hydrochloride*), AHBH3 (*A. hydrophila* injection of 10^5^ CFU/L and exposure to 300 mg/L of *berberine hydrochloride*). N per group = 10

Most of the gene expression selected in this study showed an increased trend as berberine hydrochloride concentration increased (Fig. 5). At 200 and 300 mg/L, FGA gene expression significantly increased without *A. hydrophila* injection (*P* < 0.05). At 100 mg/L without *A. hydrophila* injection and higher concentrations (200 and 300 mg/L) with *A. hydrophila* injection, FGB gene expression significantly increased (*P* < 0.05). At 200 mg/L with *A. hydrophila* injection, A2M gene expression level was significantly stimulated (*P* < 0.05). At 100 and 200 mg/L with *A. hydrophila* injection, KNG expression level significantly decreased (*P* < 0.05). The gene expression level of MMP1 significantly increased at all concentrations of berberine hydrochloride without *A. hydrophila* injection (*P* < 0.05). CASP8 showed a similar trend to MMP1. ASHG showed no significant changes in all groups.

### Survival rate and activities of selected immune enzymes

**Fig. 6:**
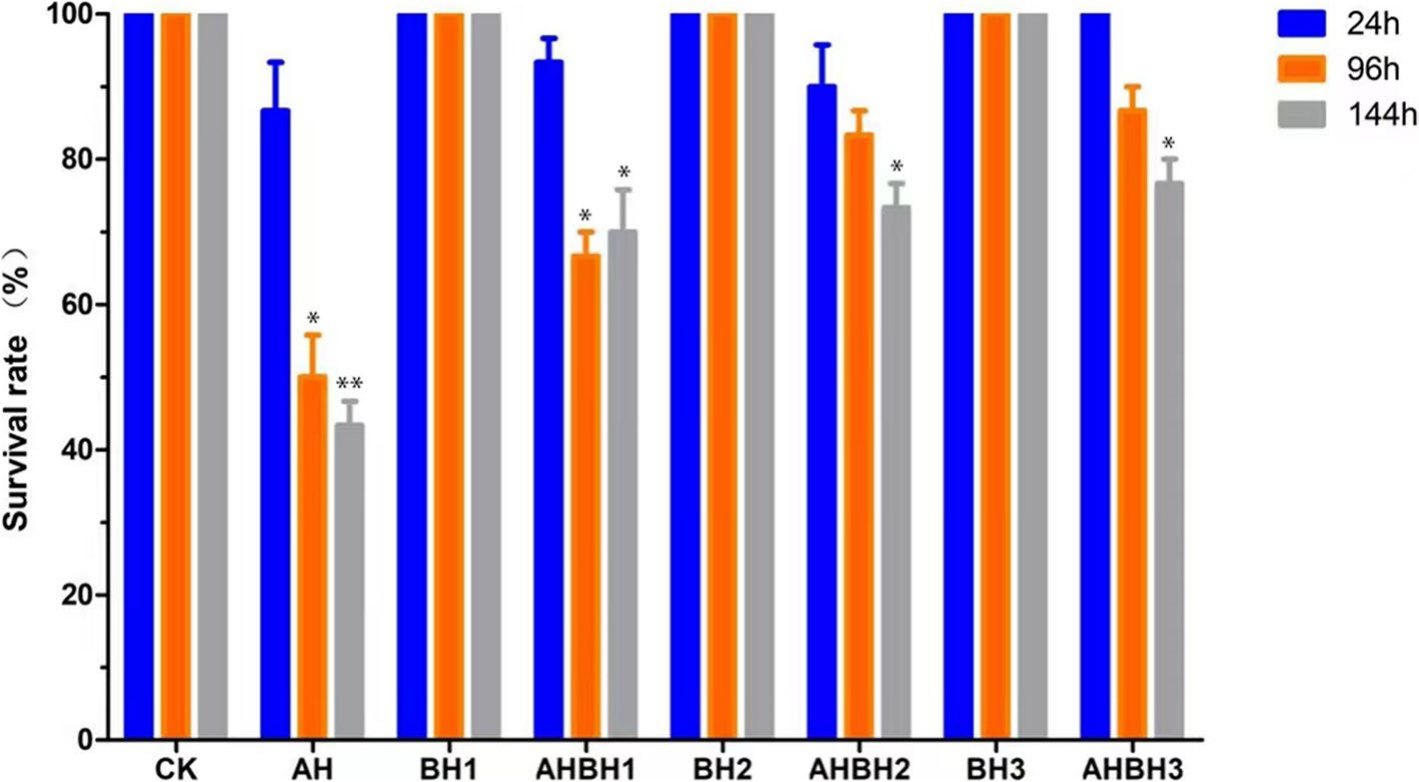
The effect of berberine hydrochloride on the activities of immune related enzymes in *Charybdis japonica.* n = 3, *P* < *0.05*

**Fig. 7:**
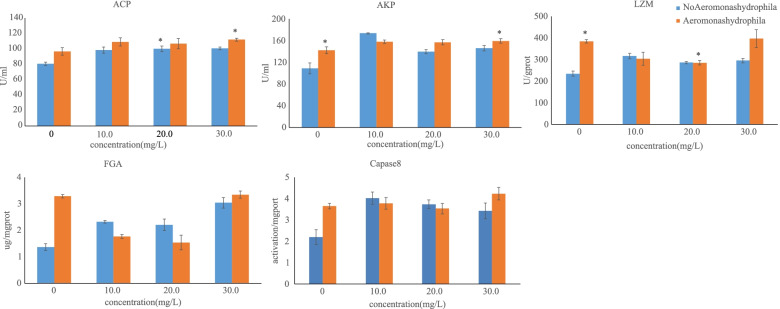
The effect of berberine hydrochloride on the gene expression of immune related enzymes in *Charybdis japonica.* n = 3, *P* < *0.05*

Between groups injected with *A. hydrophila*, the survival rate was highest at the berberine hydrochloride concentration of 300 mg/L (Fig. 6). This group showed a significant increase of the activity of LZM, caspas8, FGA, ACP and AKP in the hepatopancreas (Fig. 7). When *A. hydrophila* was added, the neutralization of 300 mg/L berberine hydrochloride maximized the activity of Caspas8, LZM, ACP and AKP in the hepatopancreas.

## Discussion

This study found that 962 genes were significantly up-regulated or down-regulated under the action of berberine hydrochloride. A number of immune-related genes were also identified, involved in the synthesis of fibrinogen, lysophospholipase, cathepsin L, chitinase, alpha-2-macroglobulin (A2M), kininogen (KNG), cathepsin L (CTSL), caspase-8 (CASP8), alkaline phosphatase (AKP), acid phosphatase (ACP) and lysozyme (LSZ). These genes play an important role in the innate immunity of crustaceans by activating specific antiviral and antibacterial immune responses. Fibrinogen is a protein with coagulative functions and is mainly synthesized by liver cells; it represents the highest coagulation factor in the plasma and is one of the primary components of blood coagulation. It is converted into fibrin through thrombin catalysis processes occurring in the circulatory system, initiating coagulation and physiological hemostasis functions, which are the primary defense reactions during direct exposure of blood to the external environment [26, 27]. Three peptide chains of fibrinogen are respectively encoded by three independent genes: FGA, FGB and FGG [28, 29]. Fibrin is an acute phase response protein, and the concentration of FGA and FGG in the plasma in the event of tissue damage, infection or inflammation can rise significantly [30]. For example, rainbow trout *Oncorhynchus mykiss* infected with *Aeromonas salmonicida* presented significant changes in FGA and FGG contents in the plasma [31]. In the present study, under the action of berberine hydrochloride, the expression of FGA, FGB, and FGG in *C. japonica* increased significantly, which in turn determined an increase in fibrinogen content and provided a strong line of defense against the pathogen.

The antimicrobial peptide Alpha2-macroglobulin (A2M) is involved in the phenoloxidase system, phagocytosis, and the antimicrobial peptide system in crabs [32]. Shrimps express large amounts of the multifunctional A2M protein in plasma and A2M is highly up-regulated after microbial infection [33]. It is known that A2M can inhibit a variety of proteases by inhibiting plasmin and kallikrein, therefore, demonstrating an anti-protease action, and it can also act as a carrier protein [34]. Additionally, it can directly decompose bacteria and fungi, inhibit the replication of bacteria or viruses, or act as an opsonin to increase the amount of phagocytosis, and even neutralize bacteria [35]. It appears that the A2M level detected in *C. japonica*’s hepatopancreas showed an increasing trend during the response to *A. hydrophila* infection, which presented in the hemolymph coagulation system and resulted in a strong defense against infection. It seems that the effector cells and inflammatory factors affect the RNA expression of MMPs simultaneously, thereby regulating their reproduction and immunity [36], specifically affecting the expression of MMP1 [37, 38].

Epithelium contributes to mesenchymal transition, and stimulates angiogenesis [39, 40], and induces growth and repair functions [40]. As a result, when the organism responds to pathogens by increasing protease activity and the pathogens in turn release exogenous proteases, A2M attaches to the exogenous proteases, removing them, preventing tissue damage. The present study showed that, under the action of berberine, the hepatopancreas infection caused by *A. hydrophila*, is counteracted by increased A2M levels that protect the tissues, while MMPs are out of balance with their inhibitors due to the breakdown of the dynamic balance of extracellular mechanisms [41, 42].

A study of the serum protein AHSG reported a correlation between AHSG polymorphisms and AHSG serum concentration levels [43, 44]. The utilization of AHSG by macrophages is essential for regulating the innate immune response in case of tissue damage and infection, and it is noteworthy that berberine hydrochloride promotes the up-regulation of AHSG in *C. japonica*. AHSG is an endogenous cation, and its increase is necessary for the inactivation of macrophages [45].

Adcy3, which encodes type 3 adenylate cyclase, participates in the cAMP signaling pathway [46]. The significant functions of the gene pathways that are up-regulated by cAMP signaling involve nutrient transport, carbohydrate metabolism, and lysosomal function, as well as those involved in signal transduction, gene transcription, and immune function [47]. cAMP is an important second messenger of intracellular signal transduction operating downstream of key metabolic media (such as ghrelin and the alpha-melanocyte stimulating hormones), and this process controls the development and function of adipose tissue and insulin secretion in the associated beta cells [48]. In fish, the combination of SNP sites in Adcy3 showed a significant effect on the resistance against *Vibrio anguillarum* [49]. It has been shown that the up-regulation of Adcy3 may activate other immune factors and promote the synthesis of other immune-related proteins.

Kallikrein kinin system (KKS) consists of two main cascades: plasminogen (HMW) and plasma kininogen (KKS). These two proteins are conserved among vertebrates and are involved in inflammatory processes. The concentration of acute C-reactive protein (CRP) in the plasma increases sharply when the body is infected or damaged. As a result of this protein, complement activation and phagocytosis processes are enhanced. Opsonization is subsequently enhanced leading to the elimination of pathogenic microorganisms and prevention of tissue damage, necrosis, and cell death [50]. Previous experiments showed that, during infections, KNG gene expression and C-reactive protein contents increased simultaneously, displaying a new type of interaction between the innate immune CRP system and the inflammatory protein kininogen (KNG) [51]. In this study, a depression of KNG was observed at berberine hydrochloride treated groups injected with *A. hydrophila* which suggested that some symptoms of the infection probably abated with the treatment of berberine hydrochloride.

Caspases can be divided in three categories based on different functions: initiator apoptotic, effector cell apoptotic and inflammatory [51]. Exogenous cell apoptosis depends on the formation of death-inducing signal complexes, which contain caspase-8 [52]. This enzyme plays a key role in the caspase-dependent apoptotic pathway. Up-regulation of the caspase-8 gene can effectively control exogenous cell apoptosis by reducing inflammation and the action of toxins and viruses [53, 54]. It is known that cadmium induces hepatopancreatic apoptosis through a mitochondrial caspase-dependent pathway, and it has been demonstrated that in this context, the activity of caspase-8 gradually increases in crabs. In this study, it was demonstrated that berberine hydrochloride could significantly increase the caspase-8 level in the hepatopancreas of C. *japonica* (Fig. 7). When injected with *A. hydrophila,* the fluctuation range within the group became larger but no significant differences were observed between different groups. It seems that berberine hydrochloride could significantly decrease the adverse effects caused by *A. hydrophila* by activating the apoptotic process.

Crabs, including *C. japonica,* do not have an adaptive immune system and depend upon non-specific immune responses. Specific enzymes, such as LZM, ACP and AKP, play an important role in crustacean immunity and are considered reliable indicators for assessing the non-specific immune status of crustaceans. LZM is produced in crustaceans by the activation of complement and phagocytes, through neutrophils and macrophages. After being secreted, it is used to eradicate pathogenic bacteria from the hemolymph and mucus [55]. AKP can enhance the recognition and phagocytosis of pathogens by modifying the structure of the surface of pathogen cells [56]. The lysosomal activity of ACP has similar functions to those of LZM [57]. In this experiment, *C. japonica* supplemented with berberine hydrochloride, with or without the injection of *A. hydrophila,* displayed a considerable increase in the activities of LZM, ACP and AKP in the hepatopancreas, suggesting a correlation between berberine hydrochloride and the activity of these specific enzymes. As *A. hydrophila* was injected and 300 mg/L of berberine hydrochloride was added at the same time, the neutralizing action of the bioactive compound maximized the activity of LZM, ACP and AKP in the hepatopancreas. At 24 h, 96 h and 144 h after injection, the survival rate of the group administered with 300 mg/L of berberine hydrochloride was the highest. It seems that after 24 h, the injection of *A. hydrophila* had little effect on enzyme activity. The results showed that berberine hydrochloride could significantly increase the immune ability and improve the survival rate of *C. japonica* when *A. hydrophila* was injected. At 300 mg/L, it seems to be the best effects on most of the parameters and at 100 mg/L the effects of berberine hydrochloride was obvious and sufficient for survival rate after 144 h.

In conclusion, this is the first report of transcriptome analysis of related immune gene responses under the action of berberine hydrochloride in crustaceans. In particular, LZM, ACP, and AKP were revealed as key enzymes playing a positive role in the immune response caused by berberine hydrochloride. In *C. japonica* the up-regulation of immune related genes—FGA, AHSG, MPP1, KNG, A2M, FGB and FGG—was simultaneously stimulated by berberine hydrochloride. Bioinformatics analysis showed that these genes may activate the immune complement system in the hemolymph. These findings will contribute to improving the understanding of the molecular mechanisms related to berberine hydrochloride in crustaceans, and to the development and application of treatments using this bioactive compound in aquaculture.

## Data Availability

The datasets presented in this study can be found in online repositories. The names of the repository/repositories and accession number(s) can be found below: https://www.ncbi.nlm.nih.gov/, SUB 11,219,827, Bio project accession: PRJNA819489.
